# Dietary sodium acetate and sodium butyrate improve high-carbohydrate diet utilization by regulating gut microbiota, liver lipid metabolism, oxidative stress, and inflammation in largemouth bass (*Micropterus salmoides*)

**DOI:** 10.1186/s40104-024-01009-4

**Published:** 2024-04-03

**Authors:** Qiao Liu, Liangshun Cheng, Maozhu Wang, Lianfeng Shen, Chengxian Zhang, Jin Mu, Yifan Hu, Yihui Yang, Kuo He, Haoxiao Yan, Liulan Zhao, Song Yang

**Affiliations:** College of Animal Science and Technology, Sichuan Agricultural University, Chengdu, 611130 Sichuan China

**Keywords:** High carbohydrate diet, Intestinal microbiota, Largemouth bass, Lipid deposition, Sodium acetate, Sodium butyrate

## Abstract

**Background:**

Adequate level of carbohydrates in aquafeeds help to conserve protein and reduce cost. However, studies have indicated that high-carbohydrate (HC) diet disrupt the homeostasis of the gut–liver axis in largemouth bass, resulting in decreased intestinal acetate and butyrate level.

**Method:**

Herein, we had concepted a set of feeding experiment to assess the effects of dietary sodium acetate (SA) and sodium butyrate (SB) on liver health and the intestinal microbiota in largemouth bass fed an HC diet. The experimental design comprised 5 isonitrogenous and isolipidic diets, including LC (9% starch), HC (18% starch), HCSA (18% starch; 2 g/kg SA), HCSB (18% starch; 2 g/kg SB), and HCSASB (18% starch; 1 g/kg SA + 1 g/kg SB). Juvenile largemouth bass with an initial body weight of 7.00 ± 0.20 g were fed on these diets for 56 d.

**Results:**

We found that dietary SA and SB reduced hepatic triglyceride accumulation by activating autophagy (*ATG101, LC3B* and *TFEB*), promoting lipolysis (*CPT1α, HSL* and *AMPKα*), and inhibiting adipogenesis (*FAS, ACCA, SCD1* and *PPARγ*). In addition, SA and SB decreased oxidative stress in the liver (*CAT, GPX1α* and *SOD1*) by activating the Keap1-Nrf2 pathway. Meanwhile, SA and SB alleviated HC-induced inflammation by downregulating the expression of pro-inflammatory factors (*IL-1β, COX2* and Hepcidin1) through the NF-κB pathway. Importantly, SA and SB increased the abundance of bacteria that produced acetic acid and butyrate (*Clostridium_sensu_stricto_1*). Combined with the KEGG analysis, the results showed that SA and SB enriched carbohydrate metabolism and amino acid metabolism pathways, thereby improving the utilization of carbohydrates. Pearson correlation analysis indicated that growth performance was closely related to hepatic lipid deposition, autophagy, antioxidant capacity, inflammation, and intestinal microbial composition.

**Conclusions:**

In conclusion, dietary SA and SB can reduce hepatic lipid deposition; and alleviate oxidative stress and inflammation in largemouth bass fed on HC diet. These beneficial effects may be due to the altered composition of the gut microbiota caused by SA and SB. The improvement effects of SB were stronger than those associated with SA.

**Supplementary Information:**

The online version contains supplementary material available at 10.1186/s40104-024-01009-4.

## Introduction

With the rapid development of intensive aquaculture mode, high-energy and low-protein feeds has become important factor to consider in sustainable and cost-effective fish farming [1]. As high-energy and low-cost substance, carbohydrates have been added to aquatic feeds to reduce cost [2]. However, due to physiological limitations, carnivorous fish generally have a low utilization rate of carbohydrates [3]. A high-carbohydrate (HC) diet can result in the disruption of fish intestinal flora and induce excessive lipid deposition in the liver, which can result in fatty liver [4, 5], a syndrome that is accompanied by oxidative stress and inflammation [6, 7]. The use of feed additives was one of the effective measures to mitigate the negative effects of the HC diet. For example, dietary betaine mitigated HC diet-induced hepatic lipid accumulation in blunt-snout bream by improving the expression of trimethylamine formation-associated microbial genes and bacterial taxa [8]. *Clostridium butyricum* cultures improved carbohydrate utilization, antioxidant capacity, and gut microbiota in largemouth bass fed an HC diet [9]. Similarly, studies in Nile tilapia found that insulin and *Bacillus amyloliquefaciens* ameliorated HC diet-induced metabolic syndrome by altering gut bacterial composition and enriching acetate-producing bacteria [10, 11]. Thus, utilizing gut microbes as a possible target to mitigate the detrimental effects of HC diets on fish may be a key issue for the continuous development and sustainable aquaculture approach.

The role of gut microbes in the formation of fatty liver has become a research focus on fish nutrition. The regulation of gut microbes and their metabolites is a novel method for the treatment of fatty liver, but the mechanism behind this is not fully understood [12]. Short-chain fatty acids (SCFAs) are generated by intestinal microorganisms through anaerobic fermentation [13]. Many studies have shown that SCFAs play important roles in alleviating liver steatosis, inflammatory damage, and oxidative stress [14–16]. For instance, SCFAs can regulate the TLR-mediated NF-κB cascade by promoting the secretion of its associated pro-inflammatory/anti-inflammatory cytokines, thereby modulating the immune response during inflammation [17].

Two important SCFAs, sodium acetate (SA) and sodium butyrate (SB), are noteworthy in this regard [18]. In mammals, both SA and SB can effectively reduce hepatic lipid deposition by inhibiting lipogenesis and promoting fatty acid β-oxidation, and they also participate in the regulation of hepatic lipid metabolism as regulatory factors [19–21]. In addition, SA and SB can restore mitochondrial respiratory function, resulting in enhancing antioxidant response of hepatocytes [22]. Thereby, SA and SB may be involved in mitigating the negative effects of HC on fish. Indeed, several relevant studies have examined the roles of SA and SB in nutrient metabolism, immune regulation, and the alteration of the intestinal microbial composition in aquatic animals. For example, studies in Nile tilapia found that the addition of 1.8 g/kg SA alleviated HC-induced metabolic disorders and intestinal inflammation [23], and reduced high-fat diet-induced hepatic lipid deposition and oxidative stress [24]. SA at 2 g/kg ameliorated HC diet-induced liver injury in eels [25]. Similarly, supplementation with 2 g/kg of SB reduced liver lipid deposition and improved the intestinal flora in largemouth bass [26]. Addition of 2 g/kg SB to the diet improved gut microbiota and alleviated inflammation in largemouth bass fed with high soybean meal diet [27]. These studies suggest that SA and SB are essential for metabolic homeostasis and physiological regulation. Therefore, SA and SB may be potential supplements for improving liver health and alleviating metabolic disorders induced by HC diets in fish. Similarly, the concentrations of SA and SB were set at 2 g/kg in this study. Interestingly, previous studies have demonstrated that HC diets can cause an intestinal microbial imbalance in largemouth bass, thereby reduce intestinal SA and SB levels [28]. However, whether the exogenous addition of SA and SB can mitigate the liver damage caused by excess carbohydrates in largemouth bass requires further investigation.

The largemouth bass is a typical carnivorous commercial fish. Numerous studies have identified appropriate nutrient requirements for juvenile largemouth bass. For example, the appropriate protein and lipid requirements for juvenile largemouth bass are 45.3% and 10%, respectively [29, 30]. Our research has shown that the appropriate carbohydrate requirement for juvenile largemouth bass is 9%. HC diets (18% starch) will lead to liver metabolite conversion, lipid deposition, oxidative stress, and disruption of gut flora structure in juvenile largemouth bass [31, 32]. Therefore, the goal of this study was to determine whether the exogenous addition of SA and SB could mitigate the damaging impact of carbohydrates on liver lipid deposition, oxidative stress, inflammatory, and the intestinal flora in largemouth bass. An additional goal was to explore the possible mechanisms behind this.

## Materials and methods

### Experimental diets

Five isonitrogenous (44% crude proteins) and isolipidic (10% crude lipids) experimental groups were as: group LC (9% starch), group HC (18% starch), group HCSA (18% starch; 2 g/kg SA), group HCSB (18% starch; 2 g/kg SB), and group HCSASB (18% starch; 1 g/kg SA + 1 g/kg SB). Carbohydrate levels (9% and 18%) and feed formulations were derived from our previous studies [33]. The added levels of SA and SB were based on previous studies where 2 g/kg of SA and SB were effective in improving liver and intestinal health in fish (Additional file 1: Table S1) [23–27, 34]. According to the principle of step-by-step enlargement, all feed ingredients were thoroughly mixed by hand, and soybean oil and distilled water were added sequentially. Finally, 3 mm and 4 mm sinking pellets were made by pelletizing machine [33]. The made pellet feed was placed in a cool and ventilated place for natural air drying and stored at −20 ºC. Detailed feed ingredient composition for each group is shown in Table 1. Dietary chemical composition was measured using standard method according to Baur and Ensminger [35].

**Table 1:** Formulation and chemical composition of the diets^1^, % dry matter

Ingredients	LC	HC	HCSA	HCSB	HCSASB
Fish meal^2^	51	51	51	51	51
Soybean oil^2^	6	6	6	6	6
Wheat gluten meal^2^	4	4	4	4	4
Extruded-soybean^2^	3	3	3	3	3
Spray dried plasma protein powder^2^	5	5	5	5	5
α-Starch^2^	9	18	18	18	18
Soy protein concentrate^2^	4.5	4.5	4.5	4.5	4.5
Microcrystalline cellulose^2^	12.5	3.5	3.3	3.3	3.3
Ca(H_2_PO_4_)_2_^2^	1.5	1.5	1.5	1.5	1.5
Choline chloride^2^	0.5	0.5	0.5	0.5	0.5
Yeast hydrolysate^2^	1	1	1	1	1
Vitamin premix^4^	1	1	1	1	1
Mineral premix^5^	1	1	1	1	1
Sodium acetate^3^	0.0	0.0	0.2	0.0	0.1
Sodium butyrate^3^	0.0	0.0	0.0	0.2	0.1
Chemical composition					
Crude protein^6^	44.57	44.60	44.18	44.35	44.70
Crude lipid^6^	10.40	10.29	10.42	10.24	10.52
Moisture^6^	8.68	8.58	8.70	8.89	8.75
Ash^6^	12.56	12.49	12.41	12.26	12.10
Crude fiber^6^	7.60	1.31	1.45	1.56	1.48
Nitrogen-free extract^7^	16.19	22.73	22.84	22.70	22.45
Carbohydrate^8^	23.79	24.04	24.29	24.26	23.93
Gross energy^9^, kJ/g dry matter	18.71	18.73	18.72	18.68	18.82

^1^LC represents a low carbohydrate diet; HC represents a high carbohydrate diet; HCSA, HCSB, and HCSASB represent a high carbohydrate diet supplemented with 2 g/kg SA, 2 g/kg SB, and a combination of 1 g/kg SA and 1 g/kg SB, respectively. SA = sodium acetate; SB = sodium butyrate

^2^Supplied by Chengdu Sanwang farming Co., Ltd. (China)

^3^Supplied by Xinao Agriculture and Animal Husbandry Development Co., Ltd. (China)

^4^Vitamin premix provided the following (mg/kg diet): vitamin A, 16,000 IU; vitamin D_3_, 8,000 IU; vitamin K_3_, 14.72; vitamin B_1_, 17.80; vitamin B_2_, 48; vitamin B_6_, 29.52; vitaminB_12_, 0.24; vitamin E, 160; vitamin C, 800; niacinamide, 79.20; calcium-pantothenate, 73.60; folic acid, 6.40; biotin, 0.64; inositol, 320; choline chloride, 1,500; L-carnitine, 100

^5^Mineral premix provided the following (mg/kg diet): Cu (CuSO_4_), 2.00; Zn (ZnSO_4_), 34.4; Mn (MnSO_4_), 6.20; Fe (FeSO_4_), 21.10; I (Ca (IO_3_) _2_), 1.63; Se (Na_2_SeO_3_), 0.18; Co (CoCl_2_), 0.24; Mg (MgSO_4_ ·H_2_O), 52.70

^6^Crude protein, crude lipid, ash, crude fiber and moisture contents were measured value

^7^Nitrogen-free extract = 100 – (moisture + crude protein + crude lipid + ash + crude fiber)

^8^Carbohydrate = nitrogen-free extract + crude fiber

^9^Gross energy was calculated by NRC (2011) contents [36]

### Fish preparation and feeding trial

Juvenile largemouth bass were acquired from Qionglai Hongbo Agricultural Co., Ltd. (Chengdu, China). The fish were acclimatized in the laboratory for two weeks prior to the feeding trial, and fed on the LC diet during acclimatization. The fish (initial weight: 7.00 ± 0.20 g) were randomly assigned to 15 tanks (5 groups, 3 replicates per group, 35 fish per replication). The tanks were 1 m in diameter with 300 L tap water after aeration. The fish were fed twice a day by satiation feeding for 8 weeks. Water temperature, DO, pH, and NH_4_^+^-N levels were kept at 25.0 ± 2 °C, 7.1 ± 0.5 mg/L, 6.7 ± 0.5, and < 0.15 mg/L, respectively, during feeding. Water temperature, DO, and pH were measured daily with a water quality monitor (AZ86031, Dongguan Hengxin Instrument Co., Ltd., China), and NH_4_^+^-N levels were measured weekly using Nesser's reagent spectrophotometry.

### Sample collection

The fish were fasted for 24 h and anesthetized with 0.1 g/L MS-222 (Sigma, USA) prior to sample. After the fish were dissected, the liver was removed, and a portion of it was fixed in 4% paraformaldehyde for sectioning (*n* = 3); the remaining portion was placed in a sterile tube and stored at −80 °C for subsequent Western blot, qPCR, and enzyme analyses. Intestinal contents were collected for microbiome analysis (*n* = 3).

### Tissue sectioning and staining

Liver tissues fixed in paraformaldehyde were dehydrated in ethanol (50%–100%), extracted with xylene, and embedded in paraffin. The samples were then cut into 5 µm-thick slices. The slices were partially stained with hematoxylin and eosin and partially stained with Oil red O. Photographs were taken using a digital slicing scanner and then quantized with Image J.

### Liver biochemical index analysis

Liver catalase (CAT, A007-1-1), total superoxide dismutase (T-SOD, A001-1-2), malondialdehyde (MDA, A003-1-2), glutathione peroxidase (GSH-PX, A005-1-2), triglyceride (TG, A110-1-1), and total cholesterol (T-CHO, A111-1-1) levels were measured following the instructions of commercially available kits (Nanjing Jiancheng Bioengineering Institute, China). The detection limits of these kits were 0.2 U/mL, 5.0 U/mL, 0.2 nmol/mL, 20 U/mL, 0.3 mmol/L and 0.1 mmol/L, respectively.

### Quantitative real-time PCR

Total RNA extraction and cDNA synthesis were performed following the instructions of an Animal Total RNA Isolation kit (Cat. No. RE-03014, Foregene, Chengdu, China) and RT Easy™ I (Cat. No. RT-01032, Foregene), respectively. The primer sequences were designed based on our previous genome and were listed in Table 2. The 2^−ΔΔCT^ technique was used to evaluate gene expression levels [37], with β-actin as the reference gene.

**Table 2:** Primer sequences used for real-time PCR analysis

Gene	Primer sequence (5′ → 3′)	T_m_, °C	Amplicon size, bp	E-value, %	*R*^2^	GenBank No.
*β-Actin*	F: AAAGGGAAATCGTGCGTGAC R: AAGGAAGGCTGGAAGAGGG	61	102	100.1	0.998	XM_020154709.1
*ACCA*	F:CACCAGGAACATATCGGACA R: ATCAATGTCGCTGTCAGTCT	63.0	184	99.8	0.996	XM_038709737.1
*FAS*	F: TGATGATAACTGGCTTCGG R: TCAAACCTGGACCCTACCT	60.6	86	100.1	0.998	XM_038735140.1
*SCD1*	F: CGCCGAAGATGATGGTGT R: GGAAGACTCGCAGTGGATTAG	63.0	153	98.7	0.986	XM_038735580.1
*PPARγ*	F: AGCAGACATCCGCCCTAA R: ACCTCGATCACGCCGTAC	64.3	160	99.7	0.989	XM_038695878.1
*CPT1α*	F: AGCCCCACCCCAACCTACCAG R: CGGCCCTCACGGAATAAACGC	65.0	81	99.9	0.995	XM_038695351.1
*HSL*	F: TACCCTCCGTGGCCTTTGACC R: CTCGTCTTCAGCCCCAGCGAT	64.0	161	98.9	0.985	XM_038725628.1
*PPARα*	F: CCACCGCAATGGTCGATATG R: TGCTGTTGATGGACTGGGAAA	58.0	156	100.9	0.991	XM_038738333.1
*Beclin1*	F: AGCAGTAGACGGCTGTATGAA R: TCTGGAATGCTTCTGTATGGA	59.5	120	100.1	0.995	XM_038704178.1
*ATG1*	F: ACCAGGACGCAAGTCACAC R: CAGCCTTGGCATCATAGTTT	58.4	161	99.2	0.988	XM_038704081.1
*ATG101*	F: ATTGCCGCTCGGAAGTT R: ATCGCTGCCAGAGTTGCT	58.4	275	98.5	0.995	XM_038709111.1
*ATG13*	F: GAAAGCAGGGACACGACTATG R: AACAACACCGACACGCACT	59.0	104	99.1	0.985	XM_038719443.1
*ATG4b*	F: CATACGACACACTTCGCTTTG R: CCATCTCCAGTCTCTGCCTAA	59.0	275	97.4	0.989	XM_038716733.1
*ATG5*	F: CGTGGAGGAGATGTGGTTTG R: TGACGACTTGGCTCTTGTGC	58.5	236	99.0	0.991	XM_038710515.1
*ATG7*	F: GACCCACTTGGTTTGCC R: GCGTCCCATATCTCCTTG	60.2	182	100.3	0.998	XM_038697785.1
*ATG16*	F: CTGGCTGCCTCCAATGACTT R: ACCTTCCCACTGTGACCCGT	58.4	186	102.5	0.987	XM_038692419.1
*LC3B*	F: TAAAAACACAAGCGGA R: TTGGTAGTATGTAAACGG	63.8	178	100.1	0.992	XM_038705174.1
*mcoln2*	F: GCTTCGTCATTCTTGTTTGCC R: CACACCGACCCACACCATT	58.0	296	98.9	0.988	XM_038715787.1
*TFEB*	F: GCCAACCTGACCATCAAACG R: CCACAGACGCCCGCAGTATA	58.5	208	99.5	0.995	XM_038696074.1
*Lamp2*	F: TGGTCACTGTGGTGTCGTCG R: AGGTGTTCCTGGAGGGGTTG	68.5	260	98.9	0.991	XM_038699731.1
*CAT*	F: GTTCCCGTCCTTCATCCACT R: CAGGCTCCAGAAGTCCCACA	60.4	142	100.2	0.996	XM_038704976.1
*GPX1α*	F: CCCTGCAATCAGTTTGGACA R: TTGGTTCAAAGCCATTCCCT	58.0	123	100.1	0.994	XM_038697220.1
*SOD1*	F: CCCCACAACAAGAATCATGC R: TCTCAGCCTTCTCGTGGA	58.0	86	97.8	0.989	XM_038708943.1
*Keap1*	F: TATTTCCGTCAGTCCCTCAG R: GGCAGCCAGCAGTTGTTC	63.0	215	99.4	0.990	XM_038713667.1
*Nrf2*	F: CTGGTCCGAGACATACGC R: CTCAGCAGACGCTCCTTC	57.5	168	98.6	0.992	XM_038720536.1
*IL-1β*	F: CGTGACTGACAGCAAAAAGAGG R: GATGCCCAGAGCCACAGTTC	59.4	210	99.6	0.994	XM_038733429.1
*COX2*	F: CACTGGGTCGTGTCACTTT R: TGATTCTCCTCCTTGCTGT	60.6	140	100.7	0.991	YP_636060.1
Hepcidin1	F: CATTCACCGGGGTGCAA R: CCTGATGTGATTTGGCATCATC	58.0	140	100.1	0.997	XM_038710826.1
*NF-κB*	F: TGATGATAACTGGCTTCGG R: TCAAACCTGGACCCTACCT	60.6	178	99.8	0.992	XM_038723088.1
*IL-10*	F: CGGCACAGAAATCCCAGAGC R:CAGCAGGCTCACAAAATAAACATCT	62.1	258	98.8	0.989	XM_038696252.1
*IKBα*	F: CCCCAACTACAGTGGACAAA R: AAGGTCAAGGAGGCAACG	55.6	158	99.7	0.994	XM_038734994.1

### Western blot

The liver tissue was lysed in protein lysis solution and then centrifuged at 12,000 × *g* for 20 min to obtain the supernatant. The target proteins were transferred to precast gels for electrophoresis for 50 min and then transferred onto PVDF membranes. The PVDF membrane was placed in bovine serum protein and soaked for 2 h. The PVDF membrane was removed, washed with TBST to remove the primary antibody, transferred to the secondary antibody culture, incubated for 1 h, and washed again with TBST. Finally, the chromogenic solution was added to the PVDF membrane and images were captured. Quantification of the target protein was performed using Image J. The antibodies used in this study were listed in Table S2.

### Intestinal microbiota analysis

After genomic DNA was obtained from the samples, the V3 + V4 region of 16S rDNA was amplified with specific primers. Sequencing libraries were constructed from the amplicons and sequenced on an Illumina platform. Once the raw reads were acquired, we separated the double-ended reads into tags, filtered the tags to produce clean tag, and then eliminated any low-quality or biologically irrelevant data. After that, clustering was done using the clean tag, and chimeric tags were eliminated to get the effective tag at the end. Following the acquisition of OTUs, OTU abundance data were calculated using the effective tag. The analytical process ordered the following steps: community function prediction, indicator species analysis, α-diversity analysis, β-diversity analysis, and species composition sub-licensing. The data were analyzed via the Omicsmart cloud platform (https://www.omicsmart.com).

### Statistical analysis

The results were expressed as Mean ± SEM. Significant difference between the LC and HC groups were analyzed using *t*-test, and differences among the HC, HCSA, HCSB, and HCSASB groups were analyzed by one-way ANOVA. An asterisk (*) indicates a significant difference between the LC and HC group (*P* < 0.05). Means without a common superscript differ significantly (*P* < 0.05). All data were analyzed with GraphPad Prism 9.1.

## Results

### Growth performance

**Fig. 1:**
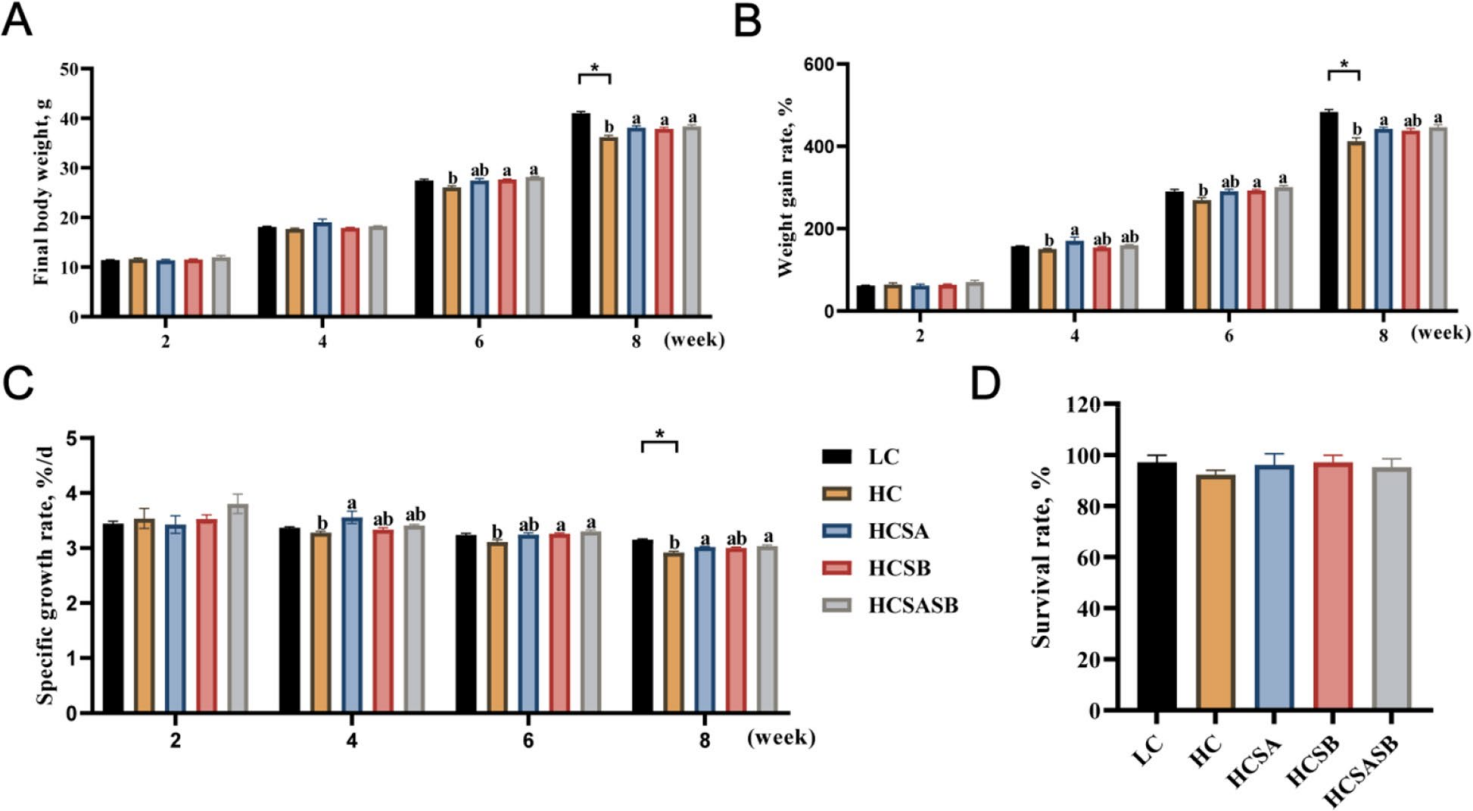
Effects of sodium acetate and sodium butyrate on growth performance of largemouth bass. **A** Final body weight (FBW). **B** Weight gain rate (WGR). **C** Specific growth rate (SGR). **D** Survival rate (SR). WGR = 100 × (final body weight − initial body weight)/initial body weight; SGR = 100 × [ln(final body weight) − ln(initial body weight)]/breeding days. Values were expressed as mean ± SEM (*n* = 3). ^a,b^Different lowercase letters indicate significant differences (*P* < 0.05). ^*^P < 0.05

This study employed the same growth experiment as in our previous work on largemouth bass [38]. After 8 weeks of the feeding trial, FBW, WGR and SGR were significantly lower in the HC group than in the LC group (*P* < 0.05) (Fig. 1A–C). At 6^th^ week, FBW, WGR and SGR in HCSB and HCSASB groups were significantly higher than those in HC group (*P* < 0.05) (Fig. 1A–C). At 8^th^ week, FBW in HCSA, HCSB and HCSASB groups were significantly higher than that in HC group, and WGR and SGR in HCSA and HCSASB groups were significantly higher than that in HC group (*P* < 0.05) (Fig. 1A–C). There was no significant difference in SR among the groups in this study (*P* > 0.05) (Fig. 1D).

### Hepatic lipid deposition

**Fig. 2:**
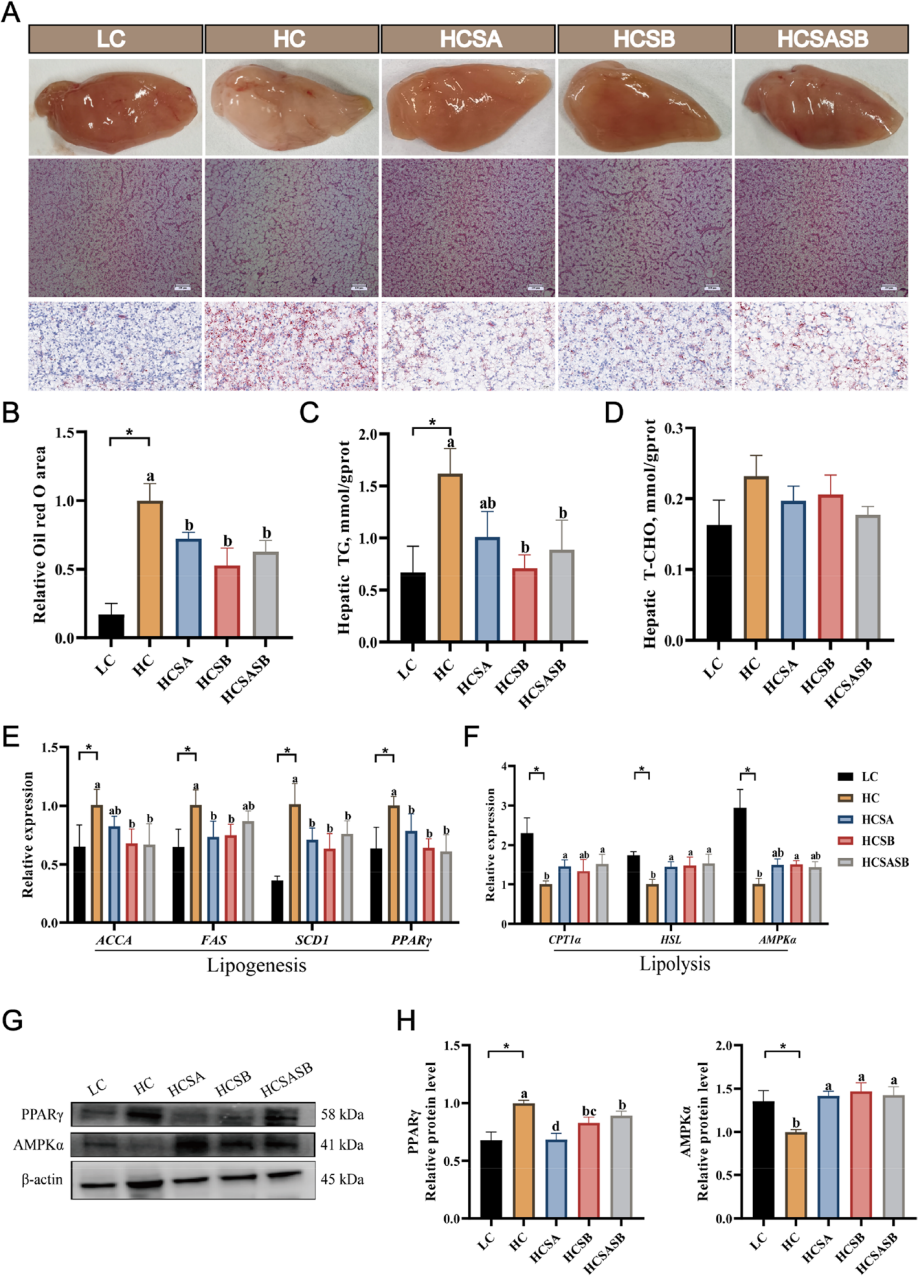
Effects of sodium acetate and sodium butyrate on the liver lipid metabolism of largemouth bass. **A** Liver morphology; HE staining of liver (scale bars, 500 μm); Oil- red O staining of liver (scale bars, 500 μm). **B** Relative Oil- red O area in liver. **C** Hepatic TG levels. **D** Hepatic T-CHO levels. **E** Expression levels of lipogenesis-related genes. **F** Expression levels of lipolysis-related genes. **G **and **H** Protein expression of PPARγ and AMPKα. Values were expressed as mean ± SEM (*n* = 6). ^a–c^Different lowercase letters indicate significant differences (*P* < 0.05). ^*^P < 0.05

The livers of the LC group appeared reddish or dark red, while those of the HC group were milky white, and those of the HCSA, HCSB, and HCSASB groups were light red with improved apparent liver color (Fig. 2A). Hematoxylin and eosin sections revealed that compared with the LC group, the livers of the HC group demonstrated severe fatty vacuolation, and most of the nuclei were displaced. However, liver fatty vacuolation was attenuated in the HCSA, HCSB and HCSASB groups (Fig. 2A). Both SA and SB attenuated HC-induced hepatic lipid deposition using Oil red O staining. (Fig. 2A and B). The biochemical indicators shown that SA and SB significantly reduced the liver triglyceride (TG) content (*P* < 0.05) (Fig. 2C), while the total cholesterol (T-CHO) content did not differ among groups (*P* > 0.05) (Fig. 2D). These results suggested that SA and SB could reduce HC-induced hepatic lipid deposition in largemouth bass.

To further investigate the mechanism by which SA and SB reduced hepatic lipid deposition, we examined the expression levels of genes involved in lipid metabolism. Compared with the LC group, the expression levels of lipid synthesis-related genes (*ACCA*, *FAS*, *SCD1*, and *PPARγ*) in the HC group were significantly increased (*P* < 0.05) (Fig. 2E), whereas the expression levels of *SCD1* and *PPARγ* were significantly depressed in the HCSA, HCSB, and HCSASB groups compared to the HC group (*P* < 0.05) (Fig. 2E). The expression level of *ACCA* in the HCSB and HCSASB groups was noticeably decreased compared to the HC group, while the expression level of *FAS* in the HCSA and HCSB groups was significantly decreased compared to the HC group (*P* < 0.05) (Fig. 2E). Likewise, the levels of key lipogenesis PPARγ proteins were strongly upregulated in the HC group (*P* < 0.05), while dietary SA and SB strongly inhibited their expression (*P* < 0.05) (Fig. 2G and H). Furthermore, the mRNA levels of lipolysis-associated genes (*CPTα*, *HSL*, and *AMPKα*) were significantly decreased in the HC group compared to the LC group (*P* < 0.05) (Fig. 2F). *CPT1α* in the HCSA and HCSASB groups was significantly lower than that in the HC group, and *AMPKα* was considerably reduced in the HCSB group compared to the LC group (*P* < 0.05) (Fig. 2F). Importantly, dietary SA and SB had markedly elevated levels of AMPKα protein expression (*P* < 0.05) (Fig. 2G and H).

### Hepatic autophagy

**Fig. 3:**
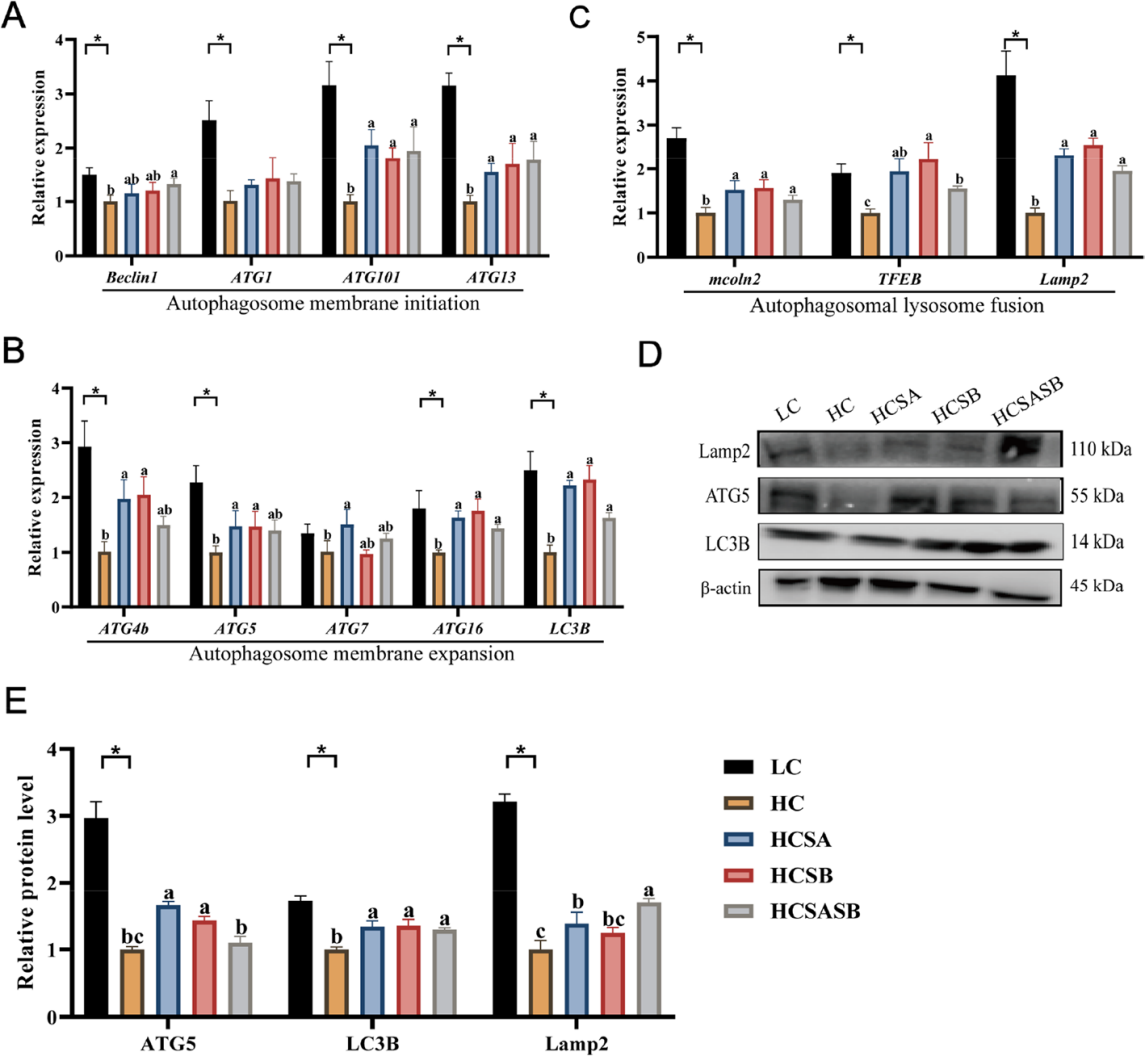
Effects of dietary sodium acetate and sodium butyrate on liver autophagy in largemouth bass. **A** Expression levels of autophagosome membrane initiation-related genes. **B** Expression levels of autophagosome membrane expansion-related genes. **C** Expression levels of autophagosomal lysosome fusion-related genes. **D**–**E** Expression levels of autophagy-related proteins. Values were expressed as mean ± SEM (*n* = 6). ^a–c^Different lowercase letters indicate significant differences (*P* < 0.05). ^*^P < 0.05

Compared with the LC group, the expression levels of autophagosome membrane initiation-related genes (*Beclin1*, *ATG1*, *ATG101*, and *ATG13*) were significantly decreased in the HC group (*P* < 0.05), whereas the expression levels of *ATG101* and *ATG13* were significantly higher in the HCSA, HCSB, and HCSASB groups than in the HC group (*P* < 0.05) (Fig. 3A). The expression levels of autophagosome membrane expansion-related genes (*ATG4b*, *ATG5*, *ATG16*, and *LC3B*) in the HC group were significantly decreased compared to the LC group (*P* < 0.05), while the expression levels of *ATG4b*, *ATG5*, *ATG16*, and *LC3B* in the HCSA and HCSB groups were markedly increased compared to those of the HC group (*P* < 0.05) (Fig. 3B). The HC group had significantly lower expression levels of autophagosome-lysosome fusion-related genes (*mcoln2*, *TFEB*, and *Lamp2*) compared to the LC group (*P* < 0.05); following SA and SB therapy, these gene expression levels were considerably increased (*P* < 0.05) (Fig. 3C). Furthermore, SA and SB significantly increased the expression levels of key autophagy-related proteins (LC3B, ATG5, and Lamp) (*P* < 0.05) (Fig. 3D and E). The results demonstrated that HC inhibited autophagy activity, but SA and SB enhanced autophagy activity, thereby improving lipid metabolism.

### Hepatic oxidative stress

**Fig. 4:**
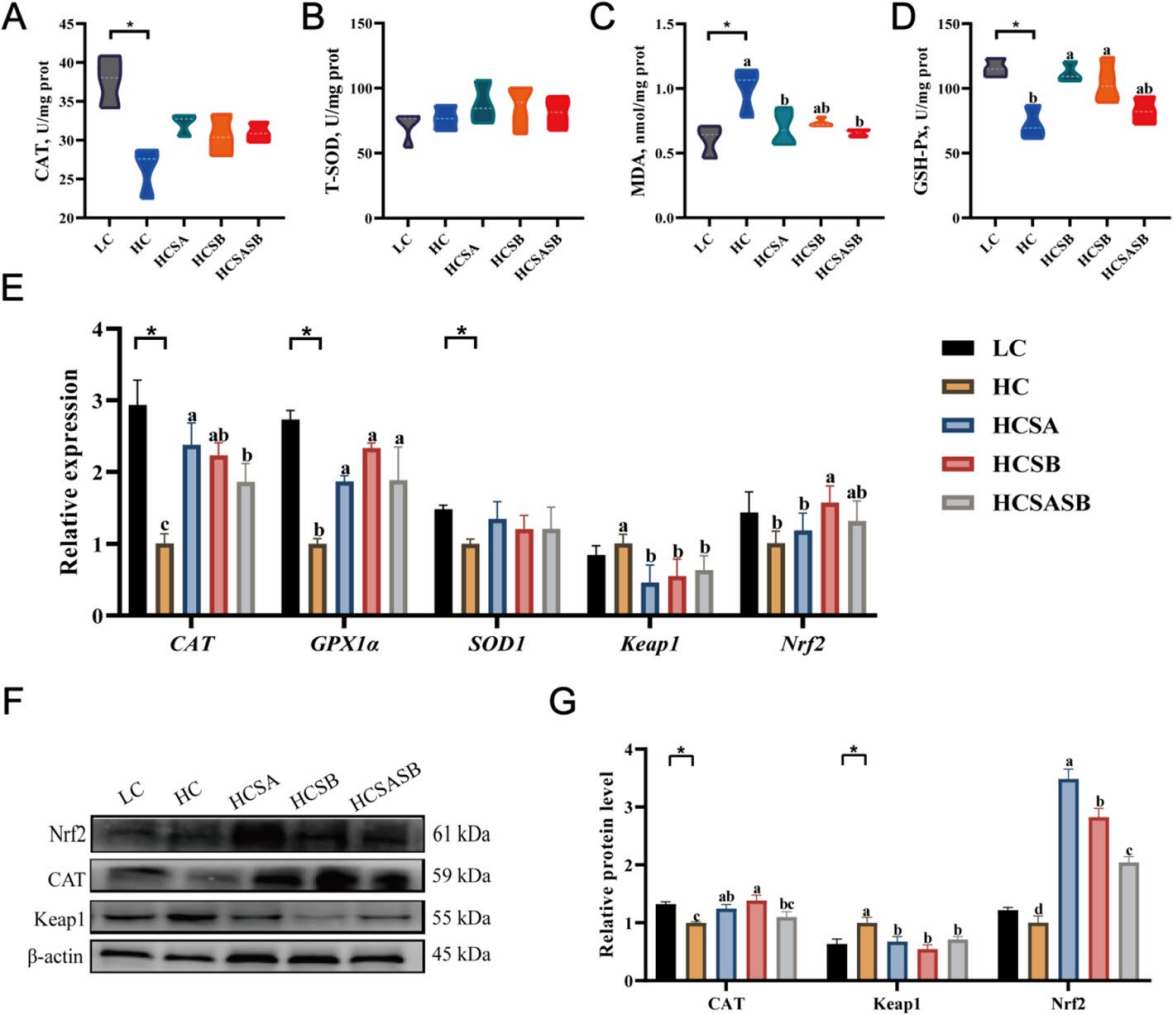
Effects of sodium acetate and sodium butyrate on the liver antioxidant capacity of largemouth bass. **A** Activities of catalase (CAT) in the liver. **B** Activities of total superoxide dismutase (T-SOD) in the liver. **C** Contents of malondialdehyde (MDA) in the liver. **D** Activities of glutathione peroxidase (GSH-Px) in the liver. **E** Expression levels of antioxidant-related genes. **F** and **G** Expression levels of antioxidant-related proteins. Values were expressed as mean ± SEM (*n* = 6). ^a–d^Different lowercase letters indicate significant differences (*P* < 0.05). ^*^P < 0.05

The MDA content in the HC group was significantly higher than in the LC group (*P* < 0.05) (Fig. 4C), although the GSH-Px activity was significantly lower (*P* < 0.05) (Fig. 4D). However, GSH-Px activity was dramatically boosted, while the MDA content was significantly lowered by SA and SB (*P* < 0.05). The activities of CAT and T-SOD were not significantly different (*P* > 0.05) (Fig. 4A and B). Meanwhile, the expression levels of antioxidant genes (*CAT*, *GPX1α*, and *SOD1*) were significantly lower in the HC than in the LC group (*P* < 0.05) (Fig. 4E). However, SA and SB significantly enhanced the transcription levels of *CAT* and *GPX1α* (*P* < 0.05) (Fig. 4E). Keap1-Nrf2 was the main regulator of antioxidant reactions; SA and SB significantly inhibited the protein expression level of Keap1 (*P* < 0.05) but increased the protein expression level of Nrf2 (*P* < 0.05) (Fig. 4F and G). Therefore, these findings revealed that SA and SB could boost antioxidant capacity in largemouth bass fed on an HC diet via activating the Keap1-Nrf2 pathway.

### Hepatic inflammation response

**Fig. 5:**
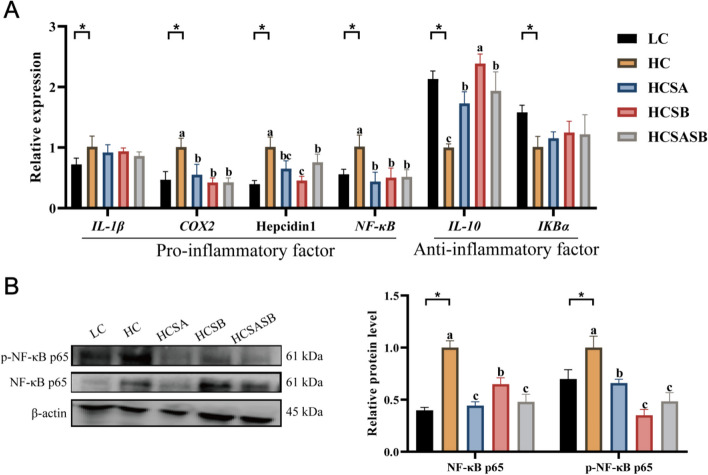
Effects of sodium acetate and sodium butyrate on liver inflammation in largemouth bass. **A** Expression levels of inflammation-related genes. **B** Expression levels of inflammation-related proteins. Values were expressed as mean ± SEM (*n* = 6). ^a–c^Different lowercase letters indicate significant differences (*P* < 0.05). ^*^P < 0.05

The HC group had considerably higher expression levels of pro-inflammatory factors (*IL-1β*, *COX2*, Hepcidin1, and *NF-κB*) compared to the LC group (*P* < 0.05) (Fig. 5A). Compared to the HC group, the expression levels of *COX2*, Hepcidin1, and *NF-κB* were significantly lower in the HCSA, HCSB, and HCSASB groups (*P* < 0.05) (Fig. 5A). However, the expression level of the anti-inflammatory factor (*IL-10*) in the HCSA, HCSB, and HCSASB groups was considerably higher than in the HC group (*P* < 0.05) (Fig. 5A). Further studies showed that SA and SB significantly inhibited total NF-κB protein and phosphorylated NF-κB protein expression (*P* < 0.05) (Fig. 5B), indicating that SA and SB could suppress HC-induced inflammation.

### Intestinal microbiome analysis

#### Diversity analysis

**Fig. 6:**
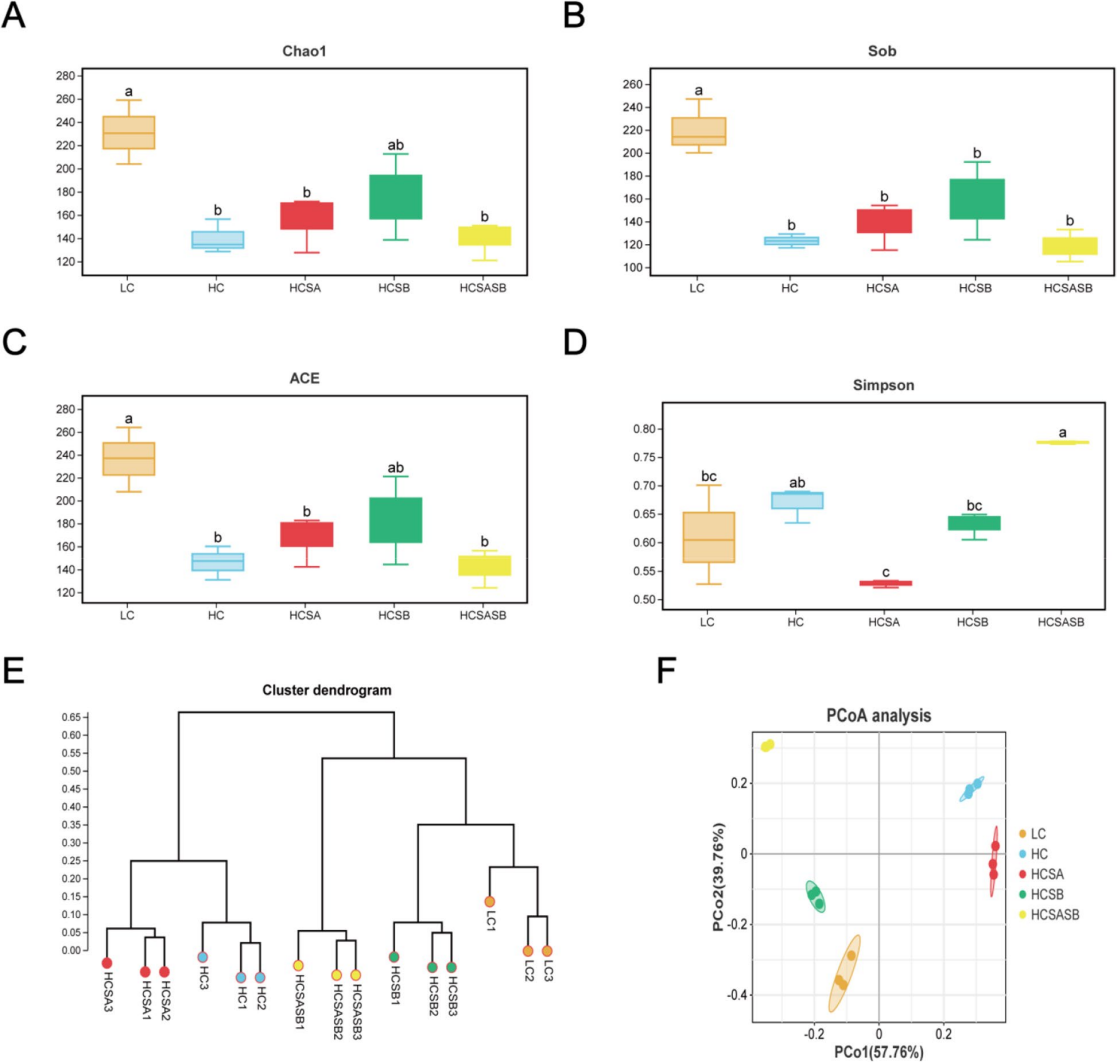
Effects of sodium acetate and sodium butyrate on the gut microbial diversity of largemouth bass. **A** Chao1 index. **B** Sob index. **C** ACE index. **D** Simpson index. **E** UPGMA hierarchical clustering based on Bray distance analysis of intestinal microbial patterns at the genus level. **E** PCoA analysis of intestinal flora at the genus level. Data were expressed as mean ± SEM (*n* = 3). ^a–c^Different lowercase letters indicate significant differences (*P* < 0.05)

The α-diversity analysis of gut microbes was presented in Fig. 6A–D. Compared to the LC group, the Chao1, Sob, and ACE indices of the HC group were significantly decreased (*P* < 0.05), while those of the HCSA, HCSB, and HCSASB groups were increased, although the difference was not observed (*P* > 0.05). Only the HCSA group’s Simpson index significantly decreased in comparison to the HC group (*P* < 0.05).

The β-diversity analysis (Fig. 6E and F) and the UPGMA classification tree (Fig. 6E) showed that the HC group and the LC, HCSB, and HCSASB groups clustered in different branches, indicating that dietary SA and SB markedly changed the intestinal flora. However, the LC and HCSB groups were initially clustered together and then clustered with the HCSASB group, suggesting that the LC, HCSB, and HCSASB groups had high similarity. The PCoA (Fig. 6F) also showed that SA and SB altered the gut microbiome composition of largemouth bass fed on an HC diet.

#### Intestinal microbial composition

**Fig. 7:**
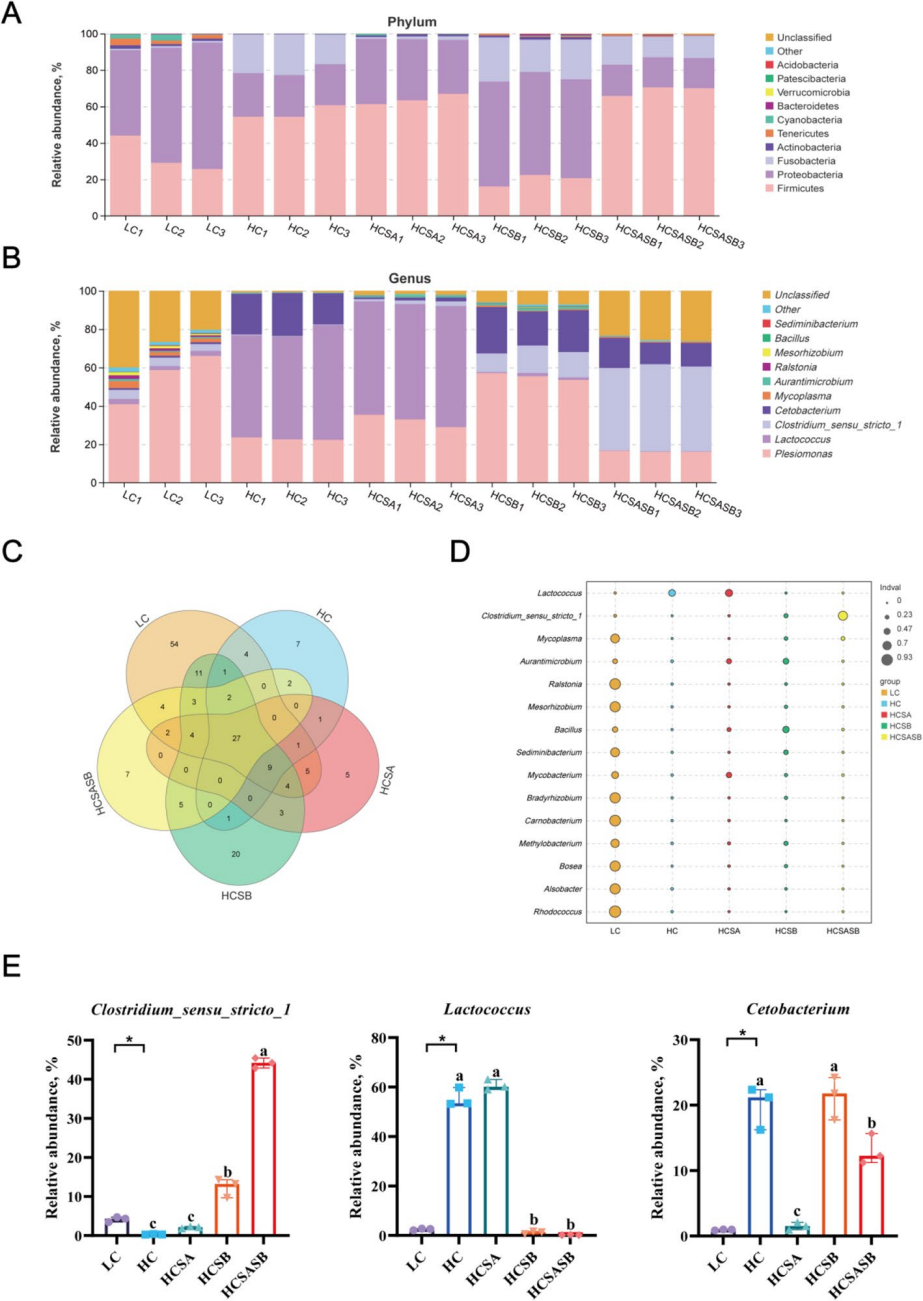
Effects of sodium acetate and sodium butyrate on the intestinal microbial composition of largemouth bass. **A** Gut flora abundance at the phylum level (Top 10). **B** Gut flora abundance at the genus level (Top 10). **C** Venn diagram of intestinal flora at the genus level. **D** Intestinal flora Indicator analysis at the genus level. **E** Significant changes in the relative abundance of intestinal microbiota observed at the genus level. Data were expressed as mean ± SEM (*n* = 3). ^a–c^Different lowercase letters indicate significant differences (*P* < 0.05). ^*^P < 0.05

The abundance of intestinal flora at the phylum level (Fig. 7A) indicated that the dominant phyla in the LC group were Proteobacteria (59%) and Firmicutes (32%). Compared to the LC group, the abundance of Proteobacteria decreased (23%) in the HC group, while the abundance of Firmicutes (56%) and Fusobacteria (20%) increased. The dominant bacteria in the HCSA group were Firmicutes (64%) and Proteobacteria (33%). The dominant phyla in the HCSB group were Proteobacteria (56%), Fusobacteria (21%), and Firmicutes (20%). The dominant phyla in the HCSASB group were Firmicutes (68%), Proteobacteria (17%), and Fusobacteria (13%). These results suggested that dietary SA and SB altered the dominant gut microbiota at the phylum level in the largemouth bass fed an HC diet.

Analysis of the gut flora at the genus level (Fig. 7B and E) indicated that the abundance of *Clostridium_sensu_stricto_1* in the HC group was remarkably lower than in the LC group, and the abundances of *Lactococcus* and *Cetobacterium* were significantly higher than those in the LC group (*P* < 0.05). The HCSB and HCSASB groups demonstrated a significant decrease in *Lactococcus* abundance and an increase in *Clostridium_sensu_stricto_1* abundance compared to the HC group (*P* < 0.05). The HCSA and HCSASB groups showed a significantly reduced abundance of *Cetobacterium* (*P* < 0.05). The Venn diagram (Fig. 7C) presented that the total effective intestinal flora comprised 393 genera, of which 131 genera were in the LC group. The HC group comprised 55 genera; there were 61 genera in the HCSA group, the HCSB group comprised 90 genera, and the HCSASB group comprised 56 genera. The five groups had 27 common genera. Indicator analysis was used to screen for marker species in each group (Fig. 7D). The results revealed that the top three biomarkers were *Sediminibacterium*, *Mesorhizobium,* and *Ralstonia* for the LC group. For the HC group, these were *Lactococcus*, *Cetobacterium*, and *Plesiomonas*. For the HCSA group, these were *Lactococcus*, *Aurantimicrobium*, and *Bacillus*. The indicators for the HCSB group were *Cetobacterium*, *Aurantimicrobium,* and *Bacillus*, and for the HCSASB group, these were *Clostridium_sensu_stricto_1*, *Cetobacterium,* and *Mycoplasma*.

### Prediction of intestinal flora function

**Fig. 8:**
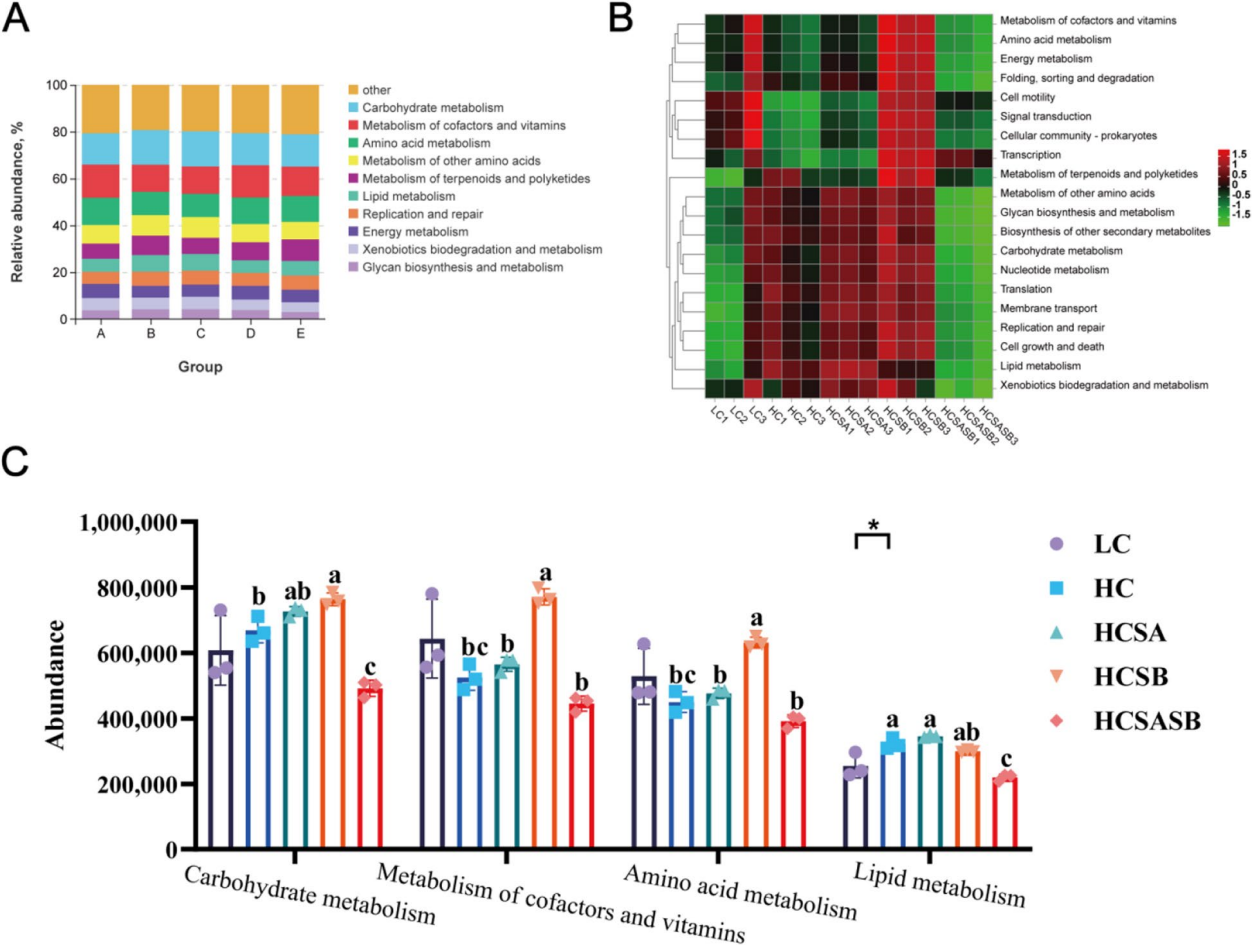
Effects of sodium acetate and sodium butyrate on the intestinal microbial KEGG functions of largemouth bass. **A** Functional stack of intestinal flora. **B** Functional clustering heat map of intestinal flora. **C** Significantly different functions of intestinal flora. Data were expressed as mean ± SEM (*n* = 3). ^a–c^Different lowercase letters indicate significant differences (*P* < 0.05). ^*^P < 0.05

Using PICRUSt to predict intestinal flora functions, the KEGG pathway analysis showed six significantly different categories, namely, metabolism, genetic information processing, cellular processes, environmental information processing, organic systems, and human diseases, as shown in the results of predictive analysis of intestinal flora functions (Fig. 8A and B). Among these categories, carbohydrate metabolism was the most abundant, followed by cofactor and vitamin metabolism, amino acid metabolism, and lipid metabolism. Therefore, a specific analysis of the four metabolic pathways was performed, and the results (Fig. 8C) indicated that compared to the LC group, lipid metabolism was significantly increased in the HC group (*P* < 0.05), while carbohydrate metabolism, cofactor and vitamin metabolism, and amino acid metabolism had no significant differences (*P* > 0.05). Carbohydrate metabolism, cofactor and vitamin metabolism, and amino acid metabolism were higher in the HCSA and HCSB groups than in the HC group (*P* < 0.05).

### Correlation between growth performance, hepatic lipid deposition, autophagy, antioxidant capacity, inflammation, and gut microbial composition

**Fig. 9:**
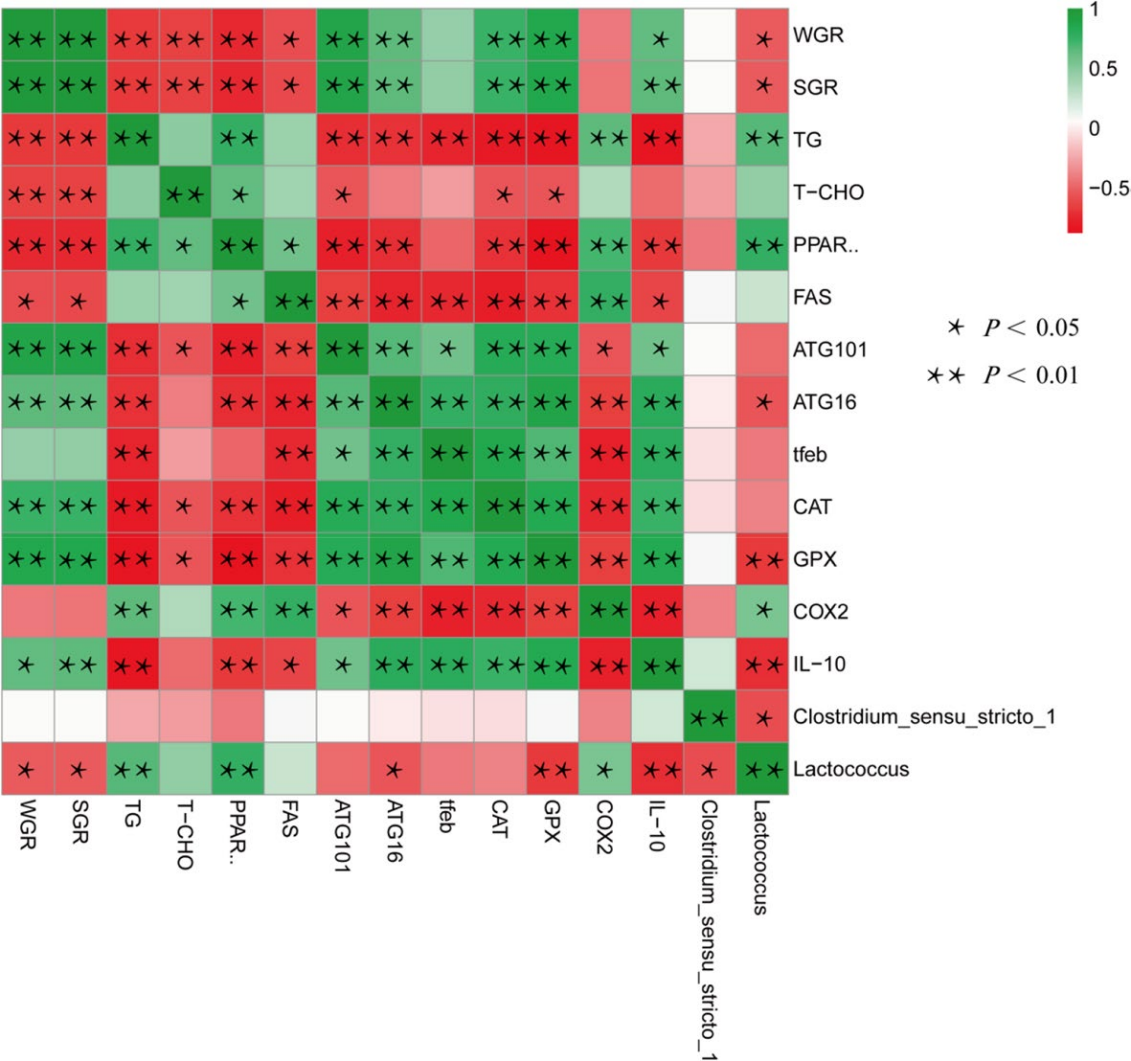
Pearson correlations between growth performance, lipid deposition, autophagy, antioxidant capacity, inflammation, and intestinal flora composition. The correlation coefficients were expressed by color intensity, ranging from green to red. ^*^P < 0.05, ^**^P < 0.01

The Pearson correlation analysis (Fig. 9) presented that growth performance was closely correlated with hepatic lipid deposition, autophagy, antioxidant capacity, inflammatory response, and intestinal microbial composition. WGR and SGR were significantly positively correlated with *ATG101*, *ATG16*, *CAT*, *GPX*, and *IL-10* mRNA expression (*P* < 0.01), and were negatively correlated with TG contents, T-CHO contents, *PPARγ*, *FAS*, and *Lactococcus* abundance (*P* < 0.01).

## Discussion

### SA and SB reduced hepatic lipid deposition by enhancing autophagy in largemouth bass fed an HC diet

Many reports have demonstrated that long-term consumption of an HC diet can cause fatty liver formation in farmed fish [8, 39, 40]. Often the formation of fatty liver can be explained by the “multiple blow model” [41]. The “first blow” is the excessive accumulation of TGs in the liver; as a result, reducing hepatic lipid deposition is the key to alleviating fatty liver. In this study, the appearance color of the liver in the HC group was milky white, implying that the HC diet caused excessive lipid deposition in the liver of largemouth bass. This was further confirmed by Oil red O staining, histological observation, and the measurement of TG content; the results were agreed with prior research [5, 42]. However, dietary sodium acetate (SA) and sodium butyrate (SB) alleviated hepatic lipid deposition in largemouth bass caused by HC feeding by inhibiting lipogenesis (*FAS*, *ACCA*, *SCD1*, and *PPARγ*) and activating the lipolysis pathway (*CPT1α*, *HSL*, and *AMPKα*). In mammals, SCFAs significantly reduce the production of fatty acid synthase in the liver by activating the UCP2-AMPK-ACCA pathway, thereby reducing lipid synthesis [43]. Moreover, SCFAs can also promote the expression of liver CPT1 promoter by inhibiting HDAC1 and enhancing promoter activity, thereby increasing CPT1 and allowing more fatty acids to enter the liver mitochondria for β-oxidation [44]. Interestingly, previous studies have shown that SA can ameliorate liver lipid deposition induced by a high-fat diet in Nile tilapia to a certain extent by activating lipid catabolism [24]. In addition, coated SB may accelerate lipid metabolism in laying hens by inhibiting liver lipogenesis enzyme activity (ACCA and FAS) and promoting liver lipolysis (AMPKα and CPT1) [45]. Therefore, SA and SB can alleviate hepatic lipid deposition in largemouth bass fed on an HC diet.

Previous research has demonstrated that autophagy is impaired in largemouth bass fed on an HC diet [33], in turn disturbing lipid metabolism [46, 47]; thus, effects of SA and SB on autophagy was investigated. Current study results have shown that SA and SB could activate autophagy, including the expression levels of autophagosome membrane initiation genes (*Beclin1*, *ATG1*, *ATG101*, and *ATG13*), autophagosome membrane expansion genes (*ATG4b*, *ATG5*, *ATG16*, and *LC3B*), and autophagosome lysosomal fusion genes (*mcoln2*, *TFEB*, and *Lamp2*). To date, there have been relatively few studies on the effects of SA and SB on liver autophagy in fish. However, a study in piglets has shown that SB induces autophagy by activating AMPK, thereby reducing H_2_O_2_-induced oxidative stress, intestinal epithelial barrier damage, and mitochondrial dysfunction [48]. Studies in mice have also shown that SB can slow the progression of osteoarthritis by restoring impaired autophagy and autophagic flux [49]. Therefore, we propose that this autophagy-promoting effect of SA and SB is maintained in the liver of HC-induced largemouth bass.

### SA and SB alleviated HC-induced hepatic oxidative stress and inflammation through Keap1-Nrf2 and the NF-κB pathway

The “second hit” in the “multi-hit model” refers to increases in oxidative stress and inflammation and is usually caused by the “first hit,” the accumulation of TGs in the liver [50]. In this study, the HC diet decreased the activities of CAT and GSH-Px in the liver, while it increased the content of MDA. This suggested that largemouth bass fed on HC diet were more susceptible to sustained oxidative stress, consistent with previous studies in golden pomfret and eels [4, 6]. The Keap1-Nrf2 pathway can regulate oxidative stress in the body. When the body is subjected to oxidative stress, the Keap1-Nrf2 signaling pathway is triggered, allowing the nuclear transcription factor Nrf2 to enter the nucleus and activate the transcription of antioxidant genes to maintain oxidation-antioxidant physiological balance [51]. In this study, both SA and SB could reduce oxidative stress by inhibiting the expression of the Keap1 protein and activating the expression of the Nrf2 protein. In addition, SA and SB increased the activities of CAT, GSH-Px, and T-SOD and decreased the content of MDA, indicating that the antioxidant activity was enhanced and that oxidative stress was reduced. It has been reported that SA and SB can restore mitochondrial respiratory function and promote mitochondrial repair by inducing mitochondrial transition to the fusion process, thus enhancing the antioxidant response of hepatocytes [16, 22]. SB is the main energy source for the intestinal epithelium; its nutritional effects can reduce oxidative stress and liver damage by increasing the concentration of glutathione [52]. Similar studies have found that the addition of SA/SB improved oxidative stress in mice through the NLRP3/Caspase-1 signaling pathway [53]. In fish, SA and SB have also been found to alleviate liver oxidative stress in Nile tilapia and largemouth bass induced by a high-fat diet [24, 26]. In summary, SA and SB can alleviate HC-induced oxidative stress in largemouth bass through the Keap1-Nrf2 signaling pathway.

Significant accumulation of TGs in the liver can induce the secretion of pro-inflammatory factors and trigger inflammation in the body [54]. In this study, we found that the HC diet activated the expression of pro-inflammatory factors (*IL-1β*, *COX2*, and Hepcidin1) and inhibited the expression of anti-inflammatory factors (*IL-10* and *IKBα*). Further research revealed that the HC diet increased the expression levels of total NF-κB protein and phosphorylated NF-κB protein. These results suggested that the HC diet induced an inflammatory response in largemouth bass. However, after treatment with SA and SB, the inflammatory response was significantly ameliorated. Recent studies have suggested that SA and SB may not only reduce the hepatitis response by improving the entero–liver axis, but also directly regulate hepatic immune cells, reduce the release of pro-inflammatory factors (*IL-1β*, *TNF-α*, and *IL-1*) in the liver, and increase the expression of anti-inflammatory factors (IL-4 and IL-10) [55, 56]. Similarly, dietary SA can upregulate immune-related genes (*TNF-α*, *TGF-β*, and *IL-8*) and improve disease resistance in common carp [57]. Dietary SB enhanced the expression of *IL-1β*, *IL-8*, and *TNF-α* in *Dicentrarchus labrax*, thereby improving anti-inflammatory function [58]. Therefore, SA and SB mitigated HC-induced inflammation through the NF-κB signaling pathway.

### SA and SB improved the intestinal microbial composition of largemouth bass fed an HC diet

Gut microbes are involved in the regulation of host nutritional metabolism, immunity, and development [59]. SCFAs produced by gut microbes can ameliorate a fatty liver condition by maintaining intestinal flora homeostasis, protecting the intestinal barrier, reducing fat deposition, and inhibiting inflammation and oxidative stress [60, 61]. The α-diversity indices (Sob, Chao1, ACE, and Shannon) can be used to assess the diversity of gut microbes. In this study, an HC diet significantly reduced Chao1, Sob, and ACE indices, indicating that gut microbial diversity was severely impaired; which was consistent with previous studies on eels and largemouth bass [62, 63]. However, SA and SB increased the α-diversity of the gut microbiome and mitigated the decrease in gut microbiome diversity caused by the HC diet. A similar study found that SA could increase the α-diversity of the gut flora and promote gut health in *Trachinotus ovatus* [64]. SB also increased the intestinal microbial diversity of Pacific white shrimp [65]. In addition, β-diversity is a comparison of diversity between different ecosystems and is measured as the rate of change of species composition between communities. A UPGMA classification tree and principal coordinate analysis (PCoA) can be used to study the similarity between samples, where the more similar samples have shorter common branches; these closer distances are also reflected in the PCoA diagram. The UPGMA classification tree and the PCoA results showed that SA and SB partially restored the intestinal microbial β-diversity altered by the HC diet.

In addition, SA and SB affected the composition of the gut microbiota. The HC diet significantly reduced the abundance of *Clostridium_sensu_stricto_1* and increased the abundance of *Cetobacterium*, which was consistent with studies for tilapia and gar [4, 10]. However, dietary SA and SB increased the abundance of *Clostridium_sensu_stricto_1*, and the abundance of *Cetobacterium* was significantly higher than that of the LC group. Studies have demonstrated that *Clostridium_sensu_stricto_1* and *Cetobacterium* can increase intestinal SA and SB concentrations [66, 67]. SA and SB play important roles in reducing hepatic lipid deposition, enhancing antioxidant capacity, and alleviating the inflammatory response [68–70]. Moreover, dietary SA and SB reduced the increase in *Lactococcus* abundance caused by the HC diet. *Lactococcus* has negative effects on host health, including inflammatory responses and the slowing of growth [71, 72]. Previous studies have shown that SA improved the gut microbiota and promoted growth performance and health in zebrafish [73]. In addition, SB can ameliorate steatohepatitis in mice fed a high-fat diet by improving intestinal flora and the intestinal barrier [74]. In conclusion, hepatic steatosis, oxidative stress, and inflammation induced by HC diet were strongly associated with gut microbial dysbiosis in largemouth bass. Fortunately, SA and SB can improve the overall health of largemouth bass fed on HC diet by restoring intestinal microbiota homeostasis.

### SA and SB enhanced metabolic pathways and improved carbohydrate tolerance in largemouth bass

In this study, the KEGG function prediction of intestinal flora showed that carbohydrate metabolism, cofactor and vitamin metabolism, and amino acid metabolism pathways were improved by SA and SB treatments, but the effect of SB was more pronounced. Studies have shown that SA and SB can be used as energy sources, providing carbon-containing precursor molecules for carbohydrate metabolism, amino acid metabolism, and cofactor metabolism and vitamin metabolism, so that they can also regulate cell metabolism by triggering signaling pathways [75]. Acetyl CoA is produced during the metabolic processing of SA and SB, and this can promote the entry of acetyl CoA into the tricarboxylic acid cycle and fat synthesis, or else promote carcass synthesis through the hydroxymethyl glutarate monoacyl CoA pathway, which promotes fat absorption and utilization [76]. In addition, SB can also accelerate the catabolic rate of sugars in animals by promoting the expression of transporter genes [77]. Recent studies have shown that dietary SA inhibited the catabolism of proteins, fats, and carbohydrates, but increased the energy supply for acetyl-CoA catabolism from dietary SA sources, ultimately increasing macronutrient deposition and promoting fish growth [78]. SB can improve the utilization of nucleotide derivatives and several common amino acids and thus increase the rate of weight gain [79]. Therefore, both SA and SB can promote the growth of largemouth bass, ameliorate the liver lipid deposition induced by excess carbohydrates, and increase the tolerance of largemouth bass to carbohydrates, possibly by improving the homeostasis and composition of the intestinal flora. In this regard, SB has an effect superior to that of SA.

## Conclusions

In this study, dietary SA and SB reduced excessive lipid deposition in the liver of largemouth bass fed on HC diet by activating autophagy, thereby alleviating oxidative stress and inflammation. Additionally, it was found that SA and SB altered the composition of the gut microbiota and increased the bacterial abundance that produce acetic acid and butyrate; consequently, which may be responsible for mitigating HC-induced liver damage and improving carbohydrate utilization. In summary, SA and SB are effective feed additives that can protect the liver health of cultured species in aquaculture practices, and the effect of SB is better than SA.

### Supplementary Information

**Additional file 1:** **Table S1.** SA and SB requirements of different fish species. **Table S2.** Antibodies used for western blot analysis.

## Abbreviations

ACCA: Acetyl-CoA carboxylase
ATG: Autophagy-related gene
Beclin1: Bcl2-associated X protein
CAT: Catalase
COX2: Cyclooxygenase 2
CPT1α: Carnitine palmitoyl transterase-1 alpha
FAS: Fatty acid synthase
GPX1α: Glutathione peroxidase-1 alpha
HSL: Hormone-sensitive lipase
IKBα: Inhibitor kappa B alpha
IL-1β: Interleukin-1β
IL-10: Interleukin-10
Keap1: Kelch like ECH-associated protein 1
LC3B: Microtubule-associated protein 1 light-chain3B
Lamp2: Lysosome-associated membrane protein 2
mcoln2: Mucolipin TRP cation channel 2
NF-κB: Nuclear factor kappa-B
Nrf2: Nuclear factor erythroid 2-related factor 2
PPAR: Peroxisome proliferator-activated receptor
SCD1: Stearoyl-CoA desaturase-1
SCFAs: Short-chain fatty acids
SOD1: Superoxide dismutase 1
TFEB: Transcription factor EB
