# Supplementary material for: Dietary sodium acetate and sodium butyrate improve high-carbohydrate diet utilization by regulating gut microbiota, liver lipid metabolism, oxidative stress, and inflammation in largemouth bass (Micropterus salmoides)
Source: J Anim Sci Biotechnol. 2024 Apr 3;15:50. doi: 10.1186/s40104-024-01009-4 (PMC10988814; doi:10.1186/s40104-024-01009-4)
Supplement: Supplementary file 1 — Additional file 1: Table S1. SA and SB requirements of different fish species. Table S2. Antibodies used for western blot analysis. [file 40104_2024_1009_MOESM1_ESM.docx]

**Table S1** SA and SB requirements of different fish species

| **Appropriate additive requirement (SA and SB, g/kg)** | **Species** | **Nutritional stress** | **Functional effect** |
| --- | --- | --- | --- |
| 1.85 (SA) | Nile tilapia  (*Oreochromis niloticus*) | High carbohydrate diet | Relieve intestinal inflammation and strengthen resistance |
| 1.85 (SA) | Nile tilapia  (*Oreochromis niloticus*) | High fat diet | Relieve liver lipid deposition and liver damage |
| 2.00 (SA) | Swamp eel  (*Monopterus albus*) | High carbohydrate diet | Enhance liver energy metabolism and alleviate liver damage |
| 1.40 (SA) | Pompano ovalis  (*Trachinotus ovatus*) | Normal diet | Promote gut health and improves microbiome |
| 1.50 (SA) | Zebrafish  (*Danio rerio*) | Normal diet | Enhance growth performance and improve intestinal health |
| 10.00 (SA) | crucian carp  (*Carassius carassius*) | Normal diet | Improve innate immunity, regulate intestinal flora, and increase disease resistance |
| 2.00 (SB) | Largemouth bass  (*Micropterus salmoides*) | High fat diet | Reduce liver lipid deposition, liver damage and improve intestinal flora |
| 2.00 (SB) | Largemouth bass  (*Micropterus salmoides*) | High soybean meal diet | Improve intestinal flora and reduce intestinal inflammation |
| 1.00-2.00 (SB) | Largemouth bass  (*Micropterus salmoides*) | Normal diet | Promote growth, improve antioxidant capacity and hypoxic stress tolerance |
| 1.00 (SB) | Grass carp  (*Ctenopharyngodon idella*) | Normal diet | Improve immunity and disease resistance |
| 2.00 (SB) | Turbot  (*Scophthalmus maximus L.*) | High soybean meal diet | Protect intestinal health and improve intestinal flora |
| 1.00 (SB) | Eel  (*Monopterus albus*) | High soybean meal diet | Improve digestive enzyme activity and intestinal health |

Relevant references were cited in the text

**Table S2** Antibodies used for western blot analysis

| **Indices** | **Host** | **Source** | **Catalog No.** | **Dilution** |
| --- | --- | --- | --- | --- |
| AMPKα | Rabbit | Immunway | YP0010 | 1:1,000 |
| PPARγ | Rabbit | Huaan Biotechnology | ET1702-57 | 1:800 |
| LC3B | Rabbit | Cell signaling | #43556 | 1:800 |
| ATG5 | Rabbit | Huaan Biotechnology | ET1611-38 | 1:1,000 |
| Lamp2 | Rabbit | Zenbio | R381078 | 1:1,000 |
| Nrf2 | Rabbit | Huaan Biotechnology | R1321-8 | 1:800 |
| Keap1 | Rabbit | abcam | EPR22664-26 | 1:1,000 |
| CAT | Rabbit | Immunway | YT0668 | 1:1,000 |
| NF-κB | Rabbit | Bioss | bs-20160R | 1:800 |
| p-NF-κB | Rabbit | Bioss | bs-5662R | 1:600 |
| β-actin | Rabbit | Zenbio | 200068-8F10 | 1:1,000 |

*AMPKα* Adenosine 5-monophosphate (AMP)-activated protein kinase alpha, *PPARγ* Peroxisome proliferator-activated receptor γ, *LC3B* Microtubule-associated protein 1 light-chain3B, *ATG5* Autophagy-related 5, *Lamp2* Lysosome-associated membrane protein 2, *Nrf2* [Nuclear factor erythroid 2-related factor 2](https://www.bing.com/ck/a?!&&p=9e124601dbe0fe05JmltdHM9MTY4ODk0NzIwMCZpZ3VpZD0yZjgzOTNiYi1kMWVjLTZkZjgtMjdlMC04MWY1ZDVlYzZjMGEmaW5zaWQ9NTU3Ng&ptn=3&hsh=3&fclid=2f8393bb-d1ec-6df8-27e0-81f5d5ec6c0a&psq=Nrf2%e7%9a%84%e8%8b%b1%e6%96%87%e5%85%a8%e7%a7%b0&u=a1aHR0cHM6Ly93d3cuc29sYXJiaW8uY29tL2dvb2RzLTc2NTQ4Lmh0bWw&ntb=1), *Keap1* Kelch-like ECH-associated protein 1, *CAT* Catalase, *NF-κB* Nuclear factor kappa-B, *p-NF-κB* Phosphorylation Nuclear factor kappa-B
